# Comprehensive analysis and implications of *Veronica persica* germination and growth traits in their invasion ecology

**DOI:** 10.1038/s41598-024-65859-8

**Published:** 2024-07-15

**Authors:** Rahmah Al-Qthanin, Asmaa M. Radwan, AbdElRaheim M. Donia, Mohamed A. Balah

**Affiliations:** 1 Department of Biology, College of Science, King Khalid University, 61413 Abha, Saudi Arabia; 2 Prince Sultan Bin-Abdul-Aziz for Environmental Research and Natural Resources Sustainability Center King Khalid University, 61413 Abha, Saudi Arabia; 3 Botany and Microbiology Department, Faculty of Science, Girls Branch, Al-Azhar University, Cairo, Egypt; 4 Medicinal and Aromatic Plants Department, Desert Research Center, Cairo, Egypt; 5 Plants Protection Department, Desert Research Center, Cairo, Egypt

**Keywords:** Environmental factors, Germination, Invasion phenology, Relative growth rate, *Veronica persica*, Ecology, Agroecology, Biodiversity, Ecophysiology

## Abstract

Invasive alien species implications in ecological threats are attributed to their unique characteristics that are linked to their invasion. *Veronica persica* (Plantaginaceae family) is an alien weed species in Egypt. Regardless of its widespread globally in various regions, the growth traits and behavior of *V. persica* remain poorly understood. The comprehensive analysis, reveals the optimal germination (Gmax) was detected at 10/20 °C, 15/20 °C, and 20/25 °C at the moderate temperature regimes. The rapid germination rate (G rate) peaked at 10/20 °C regime, with a rate of 0.376 per day. Furthermore, under stress conditions, *V. persica* has 50% germination inhibition (G_50_) and 50% of growth inhibition occurred at − 0.91 MPa and 0.75 MPa of osmotic pressure and 3225.81 ppm and 2677.1 ppm of salt stress (NaCl) respectively. The germination ranged from 6 to 9 pH, with the highest germination percentage occurring at a pH of 7 & 8, reaching 88.75% compared to the control group. There is a strong interaction effect between habitats and plant stages, the plant stages and habitats have significant effects (p ≤ 0.00) on *V. persica* growth. There was high and moderate plasticity in the response of morphological and growth features between stages. During the seedling-juvenile interval and the juvenile-flowering stages, respectively, there was a noticeable increase in both Relative Growth Rate and Net Assimilation Rate. Demographic surveys identified approximately 24 species across 11 families associated with *V. persica* in invaded areas. The Sorenson indices of qualitative index exhibited high similarity values in the invaded sites by (82.35%) compared to (72.72%) in non-invaded sites. However, interactions with native communities were reflected in lower richness, diversity, and evenness, displaying slightly higher Simpson index 1 (λ) values compared to invaded and non-invaded sites (0.043 and 0.0290) vs. (0.0207 and 0.268), in rangelands and *F. carica* orchards respectively. These results emphasize the substantially higher adaptability of *V. persica* to variable environmental conditions and abilities to invade a new community. This knowledge about invasive *V. persica* weeds germination and growth is itemized as the consistent predictive base for future invasion and informs strategic management priorities.

## Introduction

Invasive alien species pose ubiquitous threats to both agricultural and natural ecosystems^1^ and adversely impact human livelihoods^2^ due to their ecological effects and global consequences^3^. Globally, invasions by alien plants are rapidly increasing in extent and severity, leading to large-scale ecosystem degradation^4^. Understanding the forces that allow species to invade communities is critical to mitigating the impacts of invasive species^5^. Invasive species present in at least one of the two territories exhibit ‘‘winning’’ intrinsic ecophysiological features not found in native flora^6^. Successful invasions depend partly, on interactions between introduced plants and native plant communities^7^. The warmer germination temperature promoted neophyte success by increasing germination probability and germination speed, while negatively impacting these parameters in seeds of native species^8^. The distribution of invasive species is linked to environmental conditions^9^. Among habitat types, back dune patches were particularly prone to alien invasions and very efficient donors of alien plants to other patches. Salt marshes were in general very resistant to invasion but potentially acted as secondary reservoirs for some backdune alien species^10^. Invasive species display several features including fast growth rates, high reproductive rates, greater dispersal capacity, and high adaptability to a broad range of environmental conditions linked to their invasion success,^11–13^. Therefore, it is hard to control invasive and alien weeds once they are established^14^. The increase in alien plant invasions is primarily a result of the increased application of chemical fertilizers and herbicides^15^. The impacts of invasive alien species on ecosystem structures and functions are paramount for implementing appropriate management and restoration strategies^16^. Identifying the long-term trajectories of phenotypic changes in invasive species provides important clues for their appropriate management^17^. Knowledge of biological invasions is important for controlling the current distribution, understanding the causes, and mitigating the associated risks and consequences^18^.

Understanding responsible traits for promoting plant invasiveness could be important to aid in the development of adequate management strategies^19^. These traits vary from one stage to another in the life cycle of invasive species^20,21^. Functional traits may contribute to their success by enhancing niche differences or providing invasive species with competitive advantages^22^. Differences in plant morphological and physiological traits between invasive and native species are often associated with invasiveness^23^. The germination stage is a key process during the expansion of species^24^. Invasive species typically exhibit higher specific leaf area and relative growth rate^25^, as well as longer flowering and fruiting periods^26^. Relative growth rate serves as a significant determinant of the competitive ability of exotic plant species following disturbance^27^. Relative growth rate is a physiological measure that can be used as a predictor for invasiveness in disturbed habitats^28^. Trait plasticity can explain why some invasive species exhibit a better ability to establish themselves in a wide range of environments^29,30^. Higher plasticity refers to the substantial adaptability to variable environmental conditions^31^.

*Veronica L*. is the largest genus within the Plantaginaceae (Veronicaceae), boasting around 23,000 species^32,33^**.** *Veronica persica* has been identified as a weed in 27 crops across 45 countries^34^**.** It demonstrates robust germination in both light and dark conditions, thriving particularly well at 35 °C at a 2 cm depth^35^. The reproductive effort was fairly similar for both annuals and perennials of *Veronica* species, which varied in their habitats and aggressiveness as weeds^36^. The native *Veronica polita* subsp. *lilacina* placed closer to the alien *V. persica* suffered a greater decrease in fruiting success^37^. *V. persica* has special competitive effectiveness due to its polyploidy which can produce greater variations within its ecotypes^38^. This species is an alternative host for various crop pests and pathogens^39–41^. The average seed number is 50–100 seeds per plant^42^, while, it showed a mean annual decline of 18% and a half-life of 3.5 years^43^. The success of this weed is ensured by the large number of seeds and by the fact that it survives over the winter season, and is immune to frost^44^. *V. persica* has the ability to root at the nodes and can reroot following surface cultivation making it difficult to control mechanically^45,46^. *V. persica* has a high capability to produce flowering structures and a significant rate of seed production^47^. The alien farmland weeds of the genus *Veronica* exhibit adaptability to varying illumination conditions, and their asexual reproduction traits may contribute to their successful invasion^48^. Invasion of *V. cymbalaria* into areas where *V. persica* prevails is unlikely, although swift displacement of *V. cymbalaria* by *V. persica* in areas where *V. cymbalaria* is already established is also unlikely during the interference investigation^49^.

Ecological information about invasive alien species is very important for prevention methods and control efforts. In Egypt, there is inadequate knowledge about the invasive *V. persica* species seed behavior in the invaded areas. The study hypothesized that the ecological dynamics of *V. persica* will affect their behavior invasion in the new areas based on their pattern of germination and plasticity of the growth. Moreover, these results provide the base for predicting their future invasions and beyond their ecological zones. Therefore, the study aimed to test the germination and growth traits of *V. persica* under various environmental factors. Quantifying their ecological indices in their auto ecosystem communities and across different habitats to insights into the interactions between the invasive species and the native.

## Results

### Germination under fluctuating temperature regimes

The germination of *V. persica* was investigated across 36 different temperature regimes and a 12-h light/12-h dark (Table 1). The optimal temperature for *V. persica* germination was identified at 10/20 °C (93.5%), 15/20 °C (92.50%), and 20/25 °C (91.0%). Subsequently, favorable germination rates were observed at 5/20 °C and 20/25 °C (90.0% each). However, germination did not occur under extreme temperature regimes, such as 5/5 °C, 5/40 °C, and 40/40 °C regimes. The prolonged half-time of seed germination (T50) was detected at 5/35 °C by 22.89 days, however, the lowest T50 was detected at 10/15 °C by 3.87 days. The higher germination rate (G rate) was significantly noticed at 10/20 °C by 0.376 along all tested regimes.

**Table 1:** Regression equations of *Veronica persica* germination over alternating temperature.

Temperature regimes	Germination %	Equation f = a/(1 + exp(− (x − × 0)/b))	R^2^
5/5 °C	0.00^f^ ± 0.00	0.00	0.00
5/10 °C	68.50^c^ ± 6.61	1405/(1 + exp(− (x − 20.76)/4.11))	0.996
5/15 °C	88.00^b^ ± 2.83	2085.8/(1 + exp(− (x − 19.33)/4.72))	0.995
5/20 °C	90.00^ab^ ± 4.32	2213.18/(1 + exp(− (x − 18.99)/4.93))	0.995
5/25 °C	85.50^b^ ± 6.61	2201/(1 + exp(− (x − 18.94)/4.94))	0.995
5/30 °C	81.50^bc^ ± 5.51	2208.8/(1 + exp(− (x − 18.89)/4.96))	0.995
5/35 °C	24.00^e^ ± 2.83	270.5/(1 + exp(− (x − 22.89)/3.79))	0.997
5/40 °C	0.00^f^ ± 0.00	0.00	0.00
10/10 °C	58.50^d^ ± 9.57	507.16/(1 + exp(− (x − 18.83)/4.98))	0.995
10/15 °C	86.00^b^ ± 8.64	2326.28 /(1 + exp(− (x − 3.87 )/ 0.842 ))	0.996
10/20 °C	93.50^a^ ± 1.91	2621.10/(1 + exp(− (x − 4.61)/0.376))	0.994
10/25 °C	91.00^a^ ± 5.03	2419.41 /(1 + exp(− (x − 5.13)/0.509))	0.995
10/30 °C	87.00^b^ ± 4.76	2348.45/(1 + exp(− (x − 5.21)/0.401 ))	0.995
10/35 °C	28.00^e^ ± 4.32	219.41/(1 + exp(− (x − 5.22)/0.415))	0.995
10/40 °C	2.00^f^ ± 1.63	32.0/(1 + exp(− (x − 22.53)/8.27))	0.990
15/15 °C	79.50^bc^ ± 3.42	1501.4/(1 + exp(− (x − 17.75)5.66))	0.995
15/20 °C	92.50^a^ ± 1.91	2737.8/(1 + exp(− (x − 17.48)/0.818))	0.994
15/25 °C	75.50^c^ ± 3.00	2134.62/(1 + exp(− (x − 17.84)/5.79))	0.991
15/30 °C	82.00^bc^ ± 6.93	2654.26 /(1 + exp(− (x − 17.48)/5.87))	0.989
15/35 °C	36.00^d^ ± 12.11	381.1/(1 + exp(− (x − 17.83)/5.600))	0.990
15/40 °C	19.00^e^ ± 7.39	129.94 /(1 + exp(− (x − 20.57)/4.270 ))	0.993
20/20 °C	56.50^d^ ± 7.00	865.8 /(1 + exp(− (x − 17.34)/ 6.084))	0.990
20/25 °C	90.00^ab^ ± 3.79	2389.4 /(1 + exp(− (x − 17.36)/6.006))	0.989
20/30 °C	88.50^b^ ± 4.43	2608.60/(1 + exp(− (x − 17.31)/6.031))	0.989
20/35 °C	62.50^ cd^ ± 7.55	2137.18/(1 + exp(− (x− 17.61)/5.75))	0.989
20/40 °C	32.00^e^ ± 5.89	815.60 /(1 + exp(− (x − 18.55)/5.233))	0.991
25/25 °C	55.50^d^ ± 9.57	1555.08/(1 + exp(− (x − 5.34)/6.064))	0.990
25/30 °C	70.50^c^ ± 3.42	1089.41/(1 + exp(− (x − 17.31)/6.087))	0.990
25/35 °C	73.50^c^ ± 5.74	1161.53/(1 + exp(− (x − 17.35)/6.024))	0.989
25/40 °C	15.00^f^ ± 6.22	360.07 /(1 + exp(− (x − 19.58)/4.914))	0.993
30/30 °C	24.00^e^ ± 4.00	389.94/(1 + exp(− (x − 17.934)/ 5.993))	0.991
30/35 °C	27.50^e^ ± 3.79	327.37/(1 + exp(− (x − 17.39)/6.058))	0.990
30/40 °C	32.00^e^ ± 9.09	437.39 /(1 + exp(− (x − 18.91)/5.202))	0.991
35/35 °C	13.50^f^ ± 2.52	305.06/(1 + exp(− (x − 20.995)/5.984 ))	0.995
35/40 °C	7.00^f^ ± 2.00	127.85/(1 + exp(− (x − 22.61)/4.872))	0.996
40/40 °C	0.00^f^ ± 0.00	0.00	1.000
F (p value)	118.779 (0.000)		

**Figure 1:**
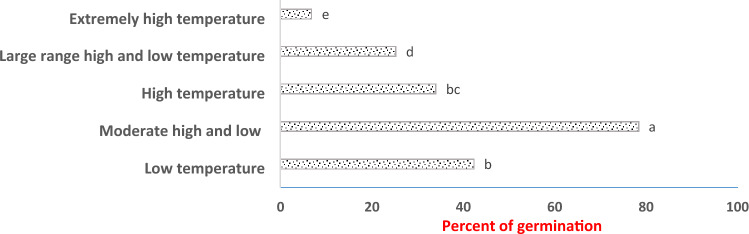
The average of germination in five consequences temperature ranges.

The average of *V. persica* germination percentage over five consequences temperatures grouped ranges was 42.33% (low-temperature regime) 78.34% (moderate-temperature regime), 34.0% (high-temperature regime), 25.25% (large high and low-temperature regime) and 6.833% (extremely high-temperature regime) respectively. The highest germination abilities recorded in *V. persica* that setting up seed germination in most temperatures and resilient adaptability in wide temperatures regimes included low, high temperature and extremely high-temperature regimes (Fig. 1).

### Germination of *V. persica* under environmental conditions

**Figure 2:**
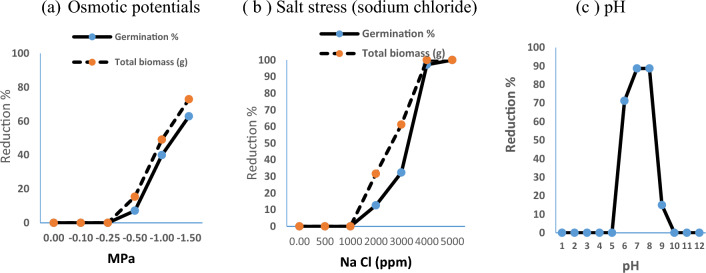
Effect of environmental stress in *V. persica* seeds germination.

The osmotic pressure in *V. persica* demonstrated a significant difference (df = 5, F = 89.07, P ≤ 0.00) in seed germination. Applying polyethylene glycol (PEG) at − 0.1 to  − 0.25 MPa, did not have any effect on seed germination. While, PEG concentrations at 0.5, − 1 and − 1.5 MPa produced a reduction percentage reached 7.14, 40.0, and 62.85%, over the control. The 50% of the germinated seed was detected at − 0.915 MPa of osmotic potentials estimated from a three-parameter logistic model; G = 65.37/(1 + exp (− (x − 0.915)/− 0.182)) R^2^ = 0.99. The osmotic pressure effect in *V. persica* seedling growth appears significantly (df = 5, F = 2162.05, P ≤ 0.00) in total biomass fresh weights. While, there is no effect of Ψ value at − 0.1 and − 0.25 MPa in seedling growth. The effect of osmotic potential treatments at 0.5, − 1 and − 1.5 MPa resulted in a reduction increasing from 15.421, 49.14 and 72.93%, respectively over the control. The 50% inhibition of the maximum growth detected at − 0.75 MPa, estimated from the fitted model; G = 76.24/(1 + exp (− (x − 0.86)/− 0.213)) R^2^ = 0.992 (Fig. 2a).

The results also indicated a significant effect of salt on the seed germination of *V. persica* (df = 6, F = 267.284, P ≤ 0.00). At low concentrations (500 to 1000 ppm), seed germination of *V. persica* was not affected by salt stress. A sharp decline in seed germination was observed from concentrations of 2000 to 4000 ppm, with reductions of 12.67%, 32.39%, and 97.18%, respectively, compared to the control. However, at concentrations ranging from 4000 to 5000 ppm of NaCl, no germination was recorded. A three-parameter logistic model was fitted to describe the reduction in germination percentage with varying NaCl concentrations. G = 102.98/{1 + exp [− (x − 3225.8) 331.36]}, R^2^ = 0.98. The concentration causing half germination (50%) was measured at 3225.81 ppm of NaCl. Regarding the salt stress effect on seedling growth (df = 6, F = 7934.99, P ≤ 0.00), the seedling fresh weight (g) gradually decreased from 2000 to 4000 ppm of NaCl, showing reductions of 31.62% to 61.19% compared to the control treatments. The fitted model; G = 104.77/(1 + exp (− (x − 2677.19)/591.44)), with an R^2^ of 0.9850%, indicated 50% inhibition in growth at 2677.1 ppm (Fig. 2b). The role of pH in *V. persica* seed germination was investigated under laboratory conditions. The result showed significant differences in seed germination (df = 10, F = 942.76, P ≤ 0.00). The suitable pH start from 6, 7, 8 and 9 with a mean germination percentage ranging from 71.25, 88.75, 88.75 and 15.0%. Unfortunately, no germination occurs at the lowest pH (1, up to 5) and the highest pH (10, 11, and 12). The pH value for half germination (G_50_), estimated from the fitted model: G = 37.67/(1 + exp (− (x − 5.25)/0.021)), R^2^ = 0.52, was determined to be 5.25. Moderate and slightly alkaline conditions were found to be favorable for *V. persica* germination (Fig. 2c).

### Life stages and growth analysis under natural habitats

**Figure 3:**
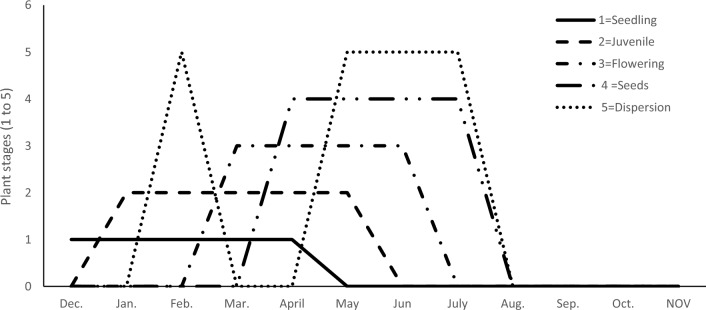
*V. persica* germination and growth life stages during the year.

The emergence of *V. persica* seeds takes place from December to May, while massive germination occurs in February and March during each year. Seedling, juvenile, flowering, seeding, and dispersion stages were distinguished with a short time of seedling and juvenile. However, the flowering stages are prolonged from March until July and occur two months after emergence. On the other side, seeding and dispersion stages are extended in the summer season (Fig. 3).

The growth traits of *V. persica* species were investigated during the growth stages in rangelands and *F. carica* orchards. The analysis revealed a significant (p ≤ 0.00) increase in total dry biomass, root weights, shoot weights, leaf mass, leaf area, and seed traits from the initial stages, reaching their optimum at the seed stage. While, the habitats have significant effects (p ≤ 0.00) on total dry biomass, root weights, shoot weights, leaves mass, and seeds respectively. The interaction effects of habitats and plant stages were significantly detected in total dry biomass, root weights, shoot weights, leaves mass, and seeds (Table 2). Regarding the phytomass accumulation at different stages for each trait, All growth traits exhibited high plasticity in their response among stages in both while these traits are lower in *F. carica* orchards than rangelands of total dry biomass, root dry weight, stem dry weight, leaves dry weight, lengths, leave area and leaves number. While, all morphological traits exhibited moderate plasticity in their response among stages in both rangelands and *F. carica* of Shoot to root ratio, leaf area ratio, leaf mass fraction, root mass fraction, stem mass fraction, specific leaf area, and specific stem length (Table 2).

**Table 2:** Plasticity index of *V. persica* and multivariate analysis of growth stages in different habitats.

Characters	Plasticity index			Variance analysis		
	Rangelands	*F. carica*			F	p value
Total dry mass	0.958	0.944	Habitats	Root (D.wt.)*	517.59	0.000
Root dry weight	0.965	0.909		Stem (D.wt.)	48.59	0.000
Stem dry weight	0.944	0.943		Leaves (D.wt.)	68.41	0.000
Leaves dry weights	0.993	0.984		total biomass (D.wt.)	10.40	0.004
Length (cm)	0.910	0.823		Seeds	265.00	0.000
Leave area (cm^2^)	0.478	0.458		Leaf area (cm 2)	0.173	0.682
number of leaves	0.946	0.934	Plant stages	Root (D.wt.)	922.57	0.000
Shoot to root ratio(g/g)	0.507	0.661		Stem (D.wt.)	1414.60	0.000
Leaf area ratio (Kg/m^2^)	0.454	0.757		Leaves (D.wt.)	92.88	0.000
Leaf mass fraction (g/g)	0.483	0.617		Total biomass (D.wt.)	478.41	0.000
Root mass fraction (g/g)	0.422	0.684		Seeds	126.29	0.000
Stem mass fraction (g/g)	0.434	0.477		Leaf area (cm 2)	240.22	0.000
Specific leaf area (m^2^/kg)	0.986	0.968	Habitats * Plant stage	Root (D.wt.)	29.67	0.000
Specific stem length (cm/kg)	0.597	0.742		Stem (D.wt.)	13.95	0.000
				Leaves (D.wt.)	32.15	0.000
				Total biomass (D.wt.)	30.63	0.000
				Seeds	99.37	0.000
				Leaves area (cm2)	0.977	0.442

*D. Wt = dry weight.

**Figure 4:**
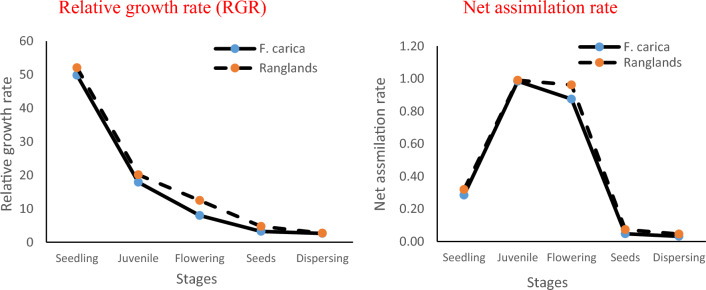
Relative growth rate and Net assimilation rate of invasive species during growth stages.

The relative growth rate (RGR) of *V. persica* was important as a key for growth measurement and competition with other plant species. The maximum RGR observed in the seedling–juvenile interval and develops slowly during the juvenile–flowering interval, then develops with a small rate at the remaining seeds and dispersing intervals, (F = 251.76, p ≤ 0.00) life stages. The net assimilation rate (NAR) is associated with photosynthetic and respiration rates. In general, there was a significant increase (F = 174, p ≤ 0.00) observed across the stages until the final stage. The net assimilation rate (NAR) was greatest in the juvenile and flowering stages but was lower in the seed and dispersing stages (Fig. 4).

### Demographic analysis of the vegetation community associated with *V. persica*

**Figure 5:**
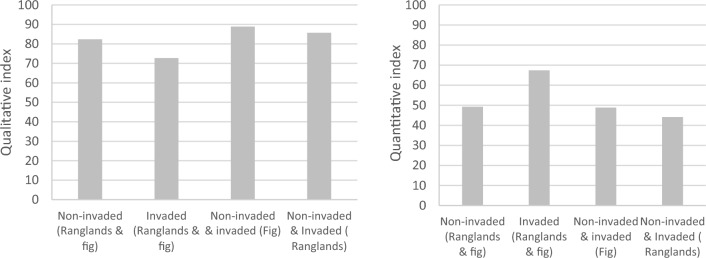
Qualitative and Quantitative indices based on species similarity in invaded and non-invaded sites of rangelands, and *F. carica* orchards.

The accompanying plants to *V. persica* species were 24 species of *Sonchus oleraceus, Capsella bursa-pastoris, Cichorium endivia, Silybum marianum, Sisymbrium irio, Chenopodium album, Convolvulus arvensis, Euphorbia peplus, Medicago indica, Medicago siculus, Pisum sativium, Vicia hirsuta, Vicia sativa, Malva parviflora, Bromus tectorum, Phalaris sp, Poa annua, Hordeum marinum, Echinochloa colonum, Lolium perenne, Cenchrus ciliaris, Cynodon dactylon, Phalaris major* and *Urtica urens* within 11 families total density (55.3 and 43.11) non invaded and (39.55 and 28.88) invaded sites of rangelands and *F. carica* respectively. The Sorenson indices of the qualitative index showed high values in the invaded sites (82.35%) compared to the non-invaded sites (72.72%) respectively. While the quantitative index showed high values in the invaded sites (67.4%) compared to the non-invaded sites (49.3%) respectively (Fig. 5).

**Figure 6:**
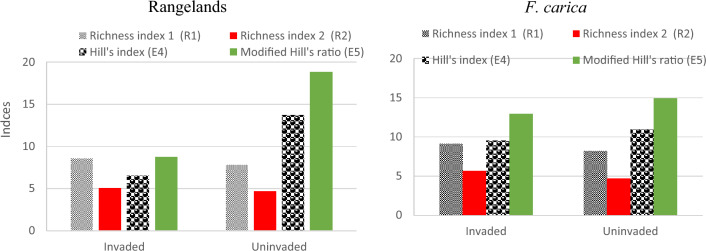
Richness index and Hill indices and ratio in invaded and non-invaded sites of rangelands, and *F. carica* orchards.

The richness index of R1 and R2 was higher in invaded sites compared to non-invaded sites of rangelands, and *F. carica* orchards. The evenness of Hill’s index (E4) and Modified Hill's ratio (E5) were higher in non-invaded areas compared to invaded areas (Fig. 6).

**Figure 7:**
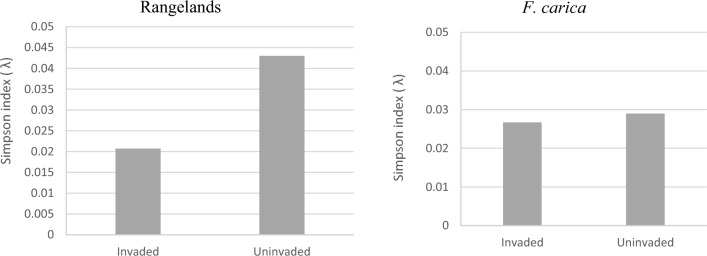
Simpson indices of diversity in invaded and non-invaded sites.

The diversity indices of the Simpson index 1 (λ) were observed slightly higher in non-invaded sites by 0.043 and 0.0290, compared to invaded sites with values of 0.0207 and 0.268 respectively in rangelands and *F. carica* (Fig. 7).

## Discussion

The limited information about invasive *V. persica* species potentials in invaded areas led to the implementation of this study to investigate its germination, and growth traits under environmental conditions as best predictors for future invasion and behavior in new habitats. The invasive species *V. persica* (Plantaginaceae) is native to Europe and parts of Asia but has spread globally as an aggressive weed and high invasion level^48^. Plant invasiveness has been correlated with seed germination traits^50^**.** The growth form, vigor, and rapid seed germination of *V. persica* are significant factors in their spreading and establishment^51^. The germination ability under a wide range of environmental conditions indicates an increased potential for invasion into non-infested areas^52^. On the basis of findings, *V. persica* seed germination preference was in moderate temperature regimes followed by low, and high-temperature regimes, consequently, their germination is limited in both widely fluctuating and extreme temperature regimes. The emergence of *V. persica* takes place from December to May, while the massive germination occurs in February and March annually. Generally, in various temperature regimes, the species exhibits high values and abilities to achieve maximum germination (G_max_) have a shorter time for 50% germination (T_50_), and a rapid germination slope (Grate)^53,54^. Seed of *V. persica* emerged from February to November with peaks in May and September^55^. *V. persica* seeds exhibited full germination when exposed to light, whereas there was only 67% germination in the dark^56^ and there was 67% germination at alternating temperatures under a ‘safe’ green light^57^. The germination timing of *V. persica* was less specific and occurred at several periods during the autumn, spring and summer^58^**.** Temperature is an important factor affecting the successful invasion and the establishment of populations that greatly affects seed germination and plant growth^59^. The invasion success is attributed to a high germination rate^60,61^.

Under respective levels of environmental stress including osmotic pressure, salt stress and varied pH levels, *V. persica* exhibited supreme tolerance to osmotic potentials (drought), with half germination (G_50_) at − 0.915 MPa with occasionally limited ability to germinate in high osmotic pressures, this osmotic potential presented 50% of growth at − 0.75 MPa. Similarly, the high salt stress concentration (5000 ppm) was not favorable for *V. persica to* germinate. It presented G_50_% at 3225.81 ppm and half growth at 2677.1 ppm. On the other side, the majority of germination was found at moderate pH levels, whereas, decreasing and increasing pH levels negatively affected *V. persica* germination. Based on these results, *V. persica* demonstrated good adaptability to germinate and establish seedlings in varied environmental conditions, including droughts, salt stress, and diverse pH levels. These results harmonized with the findings that *V. persica* Poir germinated well in both light and dark conditions^35^. In the field*, V. persica* germination was high (≥ 10%), affected by the date and depth of cultivation^62^. The presence of invasive weeds in diverse geographical regions has a considerable impact on both biodiversity and the ecological system^63^. The growth pattern of *V. persica* was recorded based on the high relative growth rate (RGR), net assimilation rate (NAR), leaf area ratio (LAR), and specific leaf area (SLA) which reflect high dry mass accumulation coefficient during the developmental growth. These traits give *V. persica* a better competitive ability as compared with the native species. Seedling relative growth rate analysis is a powerful tool for understanding life-history traits^28^. The growth form, vigor and extent of adventitious root production and rapid seed germination of *V. persica* were considered to be significant factors in their spreading and establishment^64^. Under a shaded environment, *V. persica* decreased its leaf mass per unit area (LMA) to minimize the carbon costs associated with photosynthesis and allocate more carbon for individual growth^65^. *V. persica* contributes to population recruitment through the vigorous growth of its adventitious roots^64^. Growth of *V. persica* is strongly suppressed in shade^66^. The high RGR of invasive species is advantageous initially when seedlings experience little or no competition and/or herbivory^67^. High RGR associated with opportunistic resource acquisition and increased root allocation to survive summer drought may be critical for the success of plant invaders in regions with Mediterranean climates^68^. Invasiveness is strongly related to leaf traits and fast relative growth rate that is associated with rapid carbon capture^69^. The rates of relative growth, shoots/roots ratio, leaf area ratio, and leaf mass fraction are influenced by various stages and habitats^70^. Meanwhile, *V. persica* has a high plasticity index among growth stages that reflects strong phenology traits and adaptabilities to harsh environmental conditions^71^. Alien species have strong adaptability to new habitats^72^. There is a strong correlation between vegetative growth and reproductive ability that contributes a lot to the successful invasion of *V. persica*^73^.

The ecological indices in invaded and non-invaded localities revealed, A little differentiation of Simpson index 1 (λ) in rangelands and *F. carica* by 0.025 and 0.026 (non-invaded) as compared with 0.022 and 0.219 (invaded) respectively. However, the coefficient of similarity is higher within the invaded and the non-invaded crop areas. Therefore, invasion by *V. persica* can reduce species diversity with fewer values of diversity, richness and evenness indices based on the invasion force, these detrimental impacts are caused by the displacement of native species. These results align with Fischer^74^ who reported that *V. persica* is allotetraploid adapted to become a highly successful weed with a large ecological range. While their congeneric alien species (*V. hederifolia)* has already obviously declined the biodiversity in the distribution area in China^75^. Alien *Veronica* species (*V. persica*) can capture resources successfully by increasing root biomass under shade environments^76^. Invasive species own a wider range of conditions with faster germination and higher germination rates^77,78^. While, rising temperatures due to climate change are expected to interplay with biological invasions, and may enhance the spread and growth of some alien species upon arrival in new areas^8^. A changing climate may alter the likelihood of introduction or establishment, as well as modify the geographic range, environmental impacts, economic costs or management of alien species^79^. Finally, the above result confirms the significant potential of *V. persica* to germinate under environmental stress, achieving maximum germination (G_max_), shorter time for 50% germination (T_50_), and a rapid germination slope (G_rate_) in various temperature regimes. The high accumulation coefficient and plasticity of biological traits may contribute to the extended invasion of the species. Generally, there is an association between *V. persica* germination parameters and growth traits that could act as enabling forces for invasion, spread, and colonization in new areas. Therefore, it is crucial to take all possible actions to prevent their invasion into other geographical zones.

## Materials and methods

### Plant materials

Plant specimens of naturally occurring *Veronica persica* Poir. were collected from the northwestern coast of Egypt from December to June. Voucher samples (CAIH-16-4-2022-V) were characterized by Dr. Emad Abdel-Kader, a plant specialist at the Desert Research Center, according to Tâckholm^80^ and Boulos^81^. The seeds were harvested in May and cleaned before drying in paper bags at 15 °C and 15% relative humidity and then stored in sealed vials during the germination experiment at the same conditions. The climate in this region in the winter season is rainfall, and hot dry in summer. The soil analysis revealed the following composition of 86.00% sand, 8.65% silt, and 6.35% clay respectively. The soil has ranged from 8.12 to 8.3 (pH), and 2.837 and 1.339 ds/m (electrical conductivity) respectively. Additionally, the soil contained sodium (27.721–1559 meq/l), potassium (1.145–1.098 meq/l), calcium (6.39–5.99 meq/l), magnesium (5.149–4.577 meq/l), carbonate (0.432–0.361 meq/l), bicarbonate (2.205–1.66 meq/l), and chloride (6.805–6.17 meq/l) respectively, according to established soil analysis methods FAO‐UNESCO^82^.

### Germination experimental process

The investigation of *V. persica* seed germination involved subjecting to 36 alternating temperature regimes ranging from 5 to 40 °C, with increments of 5 °C. These combinations can be categorized according to Pitcairn et al**.**^83^**,** into low temperature (5/5 °C to 5/10 °C), moderately high and low temperature (5/15 °C to 5/25 °C, 10 /15 °C to 10/30 °C, 15/15 °C to 15/35 °C, 20 /25 °C to 20/35 °C, 25/25 °C to 25/30 °C), high temperature (20/40 °C, 25/35℃ to 25/40 °C, 30/30 °C to 30/35 °C, 30/40 °C), large range high and low temperature (5/30 °C to 5/40 °C, 10/35 °C to 10/40 °C, 15/40 °C), and extremely high temperature (35/35 °C to 40/40 °C), respectively**.** Twenty-five seeds were sterilized using 0.3% sodium hypochlorite and then positioned in 9 cm Petri dishes containing two layers of filter paper moistened with distilled water. Each treatment was replicated four times and then placed in the incubator to be subject to the tested alternating temperatures ranging from 5 to 40 °C under a 12/12-h light/dark. This design followed a completely randomized pattern and was repeated at least two times to minimize errors. The number of germinated seeds was recorded daily at the same time for 30 days.

### Impact of environmental stress on *V. persica* seed germination and growth.

To explore the influence of osmotic pressure on the germination and growth of *V. persica*, Polyethylene glycol (PEG) 8000 from Sigma-Aldrich, USA, was dissolved in deionized water to produce osmotic potentials of 0.0, − 0.20, − 0.40, − 0.60, − 0.80, and − 1.00 MPa. These potentials were achieved by incorporating 0.00, 99.40, 157.10, 222.20, 314.20, and 384.80 g of PEG 8000, respectively. This method follows the protocol outlined by Michel^84^ and Michel and Radcliffe^85^. To assess the impact of salt stress, NaCl solutions with concentrations of 0.0, 500.0, 1000.0, 2000.0, 3000.0, 4000.0, and 5000.0 ppm were chosen to reflect the salinity levels found in Egyptian soil^86^. Finally, to assess the impact of varied pH levels ranging from 1.0 to 12.0, the pH solution was prepared following the method outlined by Burke et al.^87^. Twenty-five sterilized seeds of *V. persica* were positioned on filter paper placed on Petri dishes, moistened with ten milliliters of the respective test solution, and then incubated at 20/10 °C (the optimum temperature regime) under a 12-h light/12-h dark cycle. Daily counts of germinated seeds were conducted for 30 days, and the results were expressed as percentages of germination. To examine their impact on seedling growth, one seedling, seven days of age, was immersed in each concentration within a tissue culture tube for a duration of two weeks, with five replications. Subsequently, the total fresh biomass weight was recorded^86^.

### Growth development of *V. persica* in invaded habitats.

The *V. persica* samples were gathered during regular visits to the invaded area and sorted at different stages to examine their growth and development. The weights, lengths and leaf area per plant were carried out on 20 tagged plants of seedling, juvenile, flowering, seeds and dispersal stages of growth and phenology according to West and Wein^88^, whereas, leaf area ratio (LAR), root/shoot ratio (R/S ratio), leaf mass fraction (LMF), root mass fraction (RMF), stem mass fraction (SMF), specific leaf area (SLA), and specific stem length (SSL) were determined. Relative growth rate (RGR) and Net assimilation rate (NAR) were measured Cornelissen et al.^89^, and Gregory^90^ respectively. The phenotypic plasticity index is according to the following formula: IPF = (value of maximum mean – value of minimum mean)/(value of maximum mean) for each trait^91^.

### Comparison of *V. persica* communities within invaded and non-invaded sites

A field survey to quantify the occurrence of *V. persica* in rangelands and *Ficus carica* (fig) orchards on the north coast of Egypt from February to August 2021 and 2022. A total number of sites 40 included were surveyed for occurrence in rangelands and *F. carica* according to Thomas^92^ and Thomas et al.^93^. The number of species weed density and frequency determined. The *V. persica* communities under rangelands and *F. carica* systems were compared using 'Sorenson’s Indices of Similarity'^94^ that allows for comparison of stability between invaded and non-invaded situations over via Qualitative index or indices of similarity obtained from = [2C/(A + B)] × 100 where C = Number of species in common, A = Total number of species in the area A and B = total number of species in the area B.Richness, diversity index and evenness were provided according to Margalef^95^, Margalef^96^, Pielous^97^, Magrurran^98^.

### Data analysis and statistics

The germination was represented as cumulative percentages and subjected to nonlinear regression models employing a three-parameter sigmoid curve using Sigma Plot® 12.5 software, under various temperature conditions according to Evans and other^99^, and Lu et al.^100^. The employed model is G % = G_max_/1 + e [(− x − T_50)_] G_rate_. Where G represents the cumulative percentage germination at time x, G_max_ is the maximum germination (%), T_50_ is the time (d) required for 50% of maximum germination and G_rate_ indicates the slope of the curve in T_50_. The germination percentage of salt stress, osmotic potential, and different pH levels data were subject to the same equation to determine 50% inhibition of the maximum germination^83^. The growth records are subject to second-order polynomial regression equations; F = (Y0 + a*X + b*X^2^ + c*X^3^) derived from the relationship between; F is the phenotypic trait value, Y0 are fixed effects of the overall intercept, (a), (b), and (c) are fixed components of the model, X is the weights variable^70^. All data from the four replications were analyzed using analysis of variance (ANOVA) in IBM SPSS Statistics 21. Where, Tukey test was employed to compare differences within each species, and statistical significance was considered at p ≤ 0.05.

###  Guidelines of material collections and studies 

All the steps of experimentation on V. persica weeds, including the collection of plant material, are in compliance with relevant Institutional, National, and International guidelines. The studies were conducted in accordance with local legislation and with permissions from our institutes and complied with the IUCN Policy Statement.

### Supplementary Information

Supplementary Information.

## Data Availability

All data generated or analysed during this study are included in this published article and its Supplementary Information file.
