# Supplementary material for: Comprehensive analysis and implications of Veronica persica germination and growth traits in their invasion ecology
Source: Sci Rep. 2024 Jul 15;14:16285. doi: 10.1038/s41598-024-65859-8 (PMC11251038; doi:10.1038/s41598-024-65859-8)
Supplement: Supplementary file 1 — Supplementary Information. [file 41598_2024_65859_MOESM1_ESM.pdf]

Table 1 Regression equations of *Veronica persica* germination over alternating temperature

Cumulative ger. Rate

Temperature regi	5/5 ^oC	5/10 ^oC	5/15 ^oC	5/20 ^oC	5/25 ^oC	5/30 ^oC	5/35 ^oC	5/40 ^oC
Days								
1	0.000	0.000	0.000	0.000	0.000	0.000	0.000	0.000
2	0.000	0.000	0.000	0.000	0.000	0.000	0.000	0.000
3	0.000	0.000	0.000	0.000	0.000	0.000	0.000	0.000
4	0.000	0.000	0.000	0.000	0.000	0.000	0.000	0.000
5	0.000	0.000	0.000	3.500	0.000	1.500	0.000	0.000
6	0.000	0.000	6.000	31.000	28.500	24.500	0.000	0.000
7	0.000	0.000	23.500	72.500	82.000	86.000	0.000	0.000
8	0.000	0.500	89.500	151.000	156.500	163.000	0.000	0.000
9	0.000	1.500	161.000	235.000	238.000	248.000	0.000	0.000
10	0.000	17.500	241.500	319.500	323.000	333.000	0.000	0.000
11	0.000	70.000	325.500	405.500	409.000	419.000	10.500	0.000
12	0.000	127.000	409.500	492.000	495.000	505.000	22.500	0.000
13	0.000	189.500	494.500	578.500	581.000	591.000	49.000	0.000
14	0.000	253.500	581.000	666.500	668.500	678.500	95.000	0.000
15	0.000	318.000	668.000	755.000	756.500	766.500	142.500	0.000
16	0.000	384.500	755.000	843.500	844.500	854.500	190.500	0.000
17	0.000	451.500	843.000	932.500	933.000	942.500	244.500	0.000
18	0.000	518.500	931.000	1022.000	1022.000	1030.500	303.000	0.000
19	0.000	586.000	1019.000	1111.500	1111.000	1118.500	366.500	0.000
20	0.000	653.500	1107.000	1201.500	1200.000	1207.000	431.500	0.000
21	0.000	721.000	1195.000	1291.500	1289.000	1296.000	499.500	0.000
22	0.000	788.500	1283.000	1381.500	1378.000	1385.000	568.500	0.000
23	0.000	856.500	1371.000	1471.500	1467.000	1474.000	637.500	0.000
24	0.000	924.500	1459.000	1561.500	1556.000	1563.000	709.000	0.000
25	0.000	993.000	1547.000	1651.500	1645.000	1652.500	780.500	0.000
26	0.000	1061.500	1635.000	1741.500	1734.500	1742.000	852.000	0.000
27	0.000	1130.000	1723.000	1831.500	1824.000	1831.500	923.500	0.000
28	0.000	1198.500	1811.000	1921.500	1913.500	1921.000	997.000	0.000
29	0.000	1267.000	1899.000	2011.500	2003.000	2010.500	1071.000	0.000
30	0.000	1335.500	1987.000	2101.500	2092.500	2100.000	1145.000	0.000

Fig (2): Effect of environmental stress in *V. persica* seeds germination

1-Osmotic pressure Polyethyleneglycole

Mpa	R1	R2	R3	R4	Average	stdev	%	R%
0.0	18.00	17.00	18.00	17.00	17.50	87.50	0.58	0.0
-0.1	18.00	17.00	17.00	18.00	17.50	87.50	0.58	0.0
-0.3	19.00	17.00	17.00	17.00	17.50	87.50	1.00	0.0
-0.5	17.00	17.00	16.00	15.00	16.25	81.25	0.96	7.1
-1.0	10.00	9.00	11.00	12.00	10.50	52.50	1.29	40.0
-1.5	6.00	7.00	8.00	5.00	6.50	32.50	1.29	62.9

Salt stress	Nacl							
Conc (ppm)	R1	R2	R3	R4	Average	stdev	%	
0.00	18.00	17.00	19.00	17.00	17.8	1.0	88.8	0.0
500	18.00	17.00	19.00	17.00	17.8	1.0	88.8	0.0
1000	18.00	17.00	19.00	17.00	17.8	1.0	88.8	0.0
2000	15.0	16.0	17.0	14.0	15.5	1.3	77.5	12.7
3000	13.0	12.0	10.0	13.0	12.0	1.4	60.0	32.4
4000	1.0	0.0	1.0	0.0	0.5	0.6	2.5	97.2
5000	0.0	0.0	0.0	0.0	0.0	0.0	0.0	100.0

PH levels		pH					
pH	R1	R2	R3	R4	Average	stdev	%
1.0	0.0	0.0	0.0	0.0	0.0	0.0	0.0
2.0	0.0	0.0	0.0	0.0	0.0	0.0	0.0
3.0	0.0	0.0	0.0	0.0	0.0	0.0	0.0
4.0	0.0	0.0	0.0	0.0	0.0	0.0	0.0
5.0	0.0	0.0	0.0	0.0	0.0	0.0	0.0
6.0	15.0	14.0	13.0	15.0	14.3	7.6	71.3
7.0	17.0	18.0	19.0	17.0	17.8	2.1	88.8
8.0	18.0	18.0	17.0	18.0	17.8	0.7	88.8
9.0	3.0	2.0	3.0	4.0	3.0	7.9	15.0
10.0	0.0	0.0	0.0	0.0	0.0	1.7	0.0
11.0	0.0	0.0	0.0	0.0	0.0	0.0	0.0
12.0	0.0	0.0	0.0	0.0	0.0	0.0	0.0

Fig 3: *V. persica* germination and growth life stages during the year

			Fresh	dry	Fresh	dry
			Root	Root	Stem	Stem
		R1	0.08	0.01	0.19	0.06
30	Seedling	R2	0.07	0.01	0.18	0.07
		R3	0.10	0.01	0.19	0.08
		R4	0.08	0.01	0.18	0.07
			0.08	0.01	0.18	0.07
		R1	0.21	0.13	1.23	0.491
60	Juvenile	R2	0.30	0.12	1.32	0.4812
		R3	0.25	0.12	1.28	0.4362
		R4	0.25	0.12	1.28	0.470
			0.25	0.12	1.28	0.470
		R1	1.22	0.33	3.49	0.943
75	Flowering	R2	1.32	0.34	3.59	0.943

		R3	1.27	0.33	3.538	0.993
		R4	1.27	0.33	3.538	0.960
			1.27	0.33	3.538	0.960
		R1	1.60	0.54	4.84	1.468
90	Fruit	R2	1.68	0.52	4.72	1.348
		R3	1.64	0.58	4.76	1.358
		R4	1.64	0.55	4.77	1.391333
			1.64	0.55	4.77	1.391333
		R1	1.60	0.54	4.84	1.468
120	Fruit dispe	R2	1.68	0.52	4.72	1.348
		R3	1.64	0.58	4.76	1.358
		R4	1.64	0.55	4.77	1.391333
			1.64	0.55	4.77	1.39

Table (2) Plasticity index of *V. persica* and multivariate analysis of growth stages in different habitats.

Plasticity index (PI)					Mean of total dry leaves/ plants			
	stages		Root	Stem	Leaves	Fruit	Total dry n	Leav aarea
	Seedling	15	0.063	0.070	0.002	0.000	0.086	1.500
	Juvenile	30	0.215	0.470	0.035	0.000	1.105	4.286
	Flowering	60	0.332	0.960	0.167	0.000	1.884	8.420
	Seeds	90	0.577	1.391	0.189	0.003	2.709	8.608
	Dispesing	120	0.699	1.533	0.190	0.007	3.005	8.600
	Plasticity index						0.971	

Fig (4) Relative growth rate and Net assimilation rate of invasive species during growth stages.

			Relative Growth Rate					
	Root	Stem	Leaves	Fruit	whole		Root	Stem
Seedling	0.197	0.002	0.000	0.000	0.080	Seedling	196.96	2.31
Juvenile	0.067	0.004	0.000	0.000	1.031	Juvenile	67.00	4.23
Flowring buds	0.034	-0.001	0.000	0.000	1.758	Flowering	33.61	-1.46
Flowering	0.013	0.000	0.000	0.000	2.528	Fruiting	12.97	-0.06
Fruit	0.276	0.145	0.020	0.000	2.750	Dispersing	275.85	144.82

			Net Assimilstion Rate				
	Root	Stem	Leaves	Fruit	whole	Root	Stem
Seedling	0.00021	0.002	0.000	0.000	0.003	0.02	0.23
Juvenile	0.00117	0.004	0.000	0.000	0.010	0.12	0.42
Flowering	0.00054	-0.001	0.000	0.000	-0.003	0.05	-0.15
Fruit	0.00003	0.001	0.000	0.068	0.000	0.00	0.07
Fruit dispesing	0.05886	0.158	0.006	0.737	0.282	5.89	15.79

Fig (5-7) Anaysis of the community associated with V. persica

Invaded Fig

		N0 of species				No =100		
		Ficus	density			Percentage of sites		Relative
No	Family	species	Non Inf.	Infected	Non Inf.	Infected	Non Inf.	Infected
1	Asteraceae	S.oleraceus	3.00	1.00	100.00	100.00	4.00	4.00
2	Brassicacea	bursa-pastc	3.00	1.00	100.00	100.00	4.00	4.00
3	Asteraceae	C.endivia.	0.82	0.00	100.00	100.00	4.00	4.00
4	Brassicacea	S. marianun	2.00	1.00	100.00	100.00	4.00	4.00
5	Brassicacea	*Sisymbriur*	2.00	1.00	100.00	100.00	4.00	4.00
6	henopodiace	*Chenopodium album*	2.16	1.00	100.00	100.00	4.00	4.00
7	onvolvulace	*C.arvensis*	2.00	2.00	100.00	100.00	4.00	4.00
8	uphorbiace	*E.peplus*	1.90	1.43	100.00	100.00	4.00	4.00
9	Fabaceae	M.indica	1.00	0.00	100.00	100.00	4.00	4.00
10	Fabaceae	M. siculus	2.40	1.17	100.00	100.00	4.00	4.00
11	Fabaceae	*sium sativu*	3.18	2.00	100.00	100.00	4.00	4.00
12	Fabaceae	V. hirsuta	2.12	2.17	100.00	100.00	4.00	4.00
13	Fabaceae	*Vicia sativa*	1.99	2.00	100.00	100.00	4.00	4.00
14	Malvaceae	M. parviflor	2.00	1.00	100.00	100.00	4.00	4.00
15	Poaceae	*B. tectorum*	2.00	1.00	100.00	100.00	4.00	4.00
16	Poaceae	Phalaris sp	2.00	1.00	100.00	100.00	4.00	4.00
17	Poaceae	*P. annua*	3.00	2.00	100.00	100.00	4.00	4.00
18	Poaceae	H.marinum	0.78	0.00	100.00	100.00	4.00	4.00
19	Poaceae	E.colonum	1.23	0.96	100.00	100.00	4.00	4.00
20	Poaceae	L. perenne	1.70	1.15	100.00	100.00	4.00	4.00
21	Poaceae	C.ciliaris	1.95	1.68	100.00	100.00	4.00	4.00
22	Poaceae	*C.dactylon*	3.00	3.00	100.00	100.00	4.00	4.00
23	Plantagin	antago maj	1.88	0.00	100.00	100.00	4.00	4.00
24	Plantagin	Veronica	0.00	4.00	100.00	100.00	4.00	4.00
25	Urticaceae	U.urens	2.00	2.00	100.00	100.00	4.00	4.00
			49.10861	33.55	2500.00	2500.00		

Invaded Ranglads

		N0 of species				No =100		
		Ranglands	density			Percentage of sites		Relative
No	Family	species	Non Inf.	Infected	Non Inf.	Infected	Non Inf.	Infected
1	Asteraceae	S.oleraceus	2.00	1.00	100.00	100.00	3.23	3.23
2	Brassicacea	bursa-pastc	3.00	1.00	100.00	100.00	3.23	3.23

3	Asteraceae	C.endivia.	1.90	1.00	100.00	100.00	3.23	3.23
4	Asteraceae	).bonariensi	1.85	0.00	100.00	100.00	3.23	3.23
5	3rassicacea	3. marianun	2.00	1.00	100.00	100.00	3.23	3.23
6	3rassicacea	*Sisymbriun*	1.94	1.00	100.00	100.00	3.23	3.23
7	ıenopodiace	*B.vulgris*	2.00	1.00	100.00	100.00	3.23	3.23
8	ıenopodiace	*Chenopodium album*	1.18	0.86	100.00	100.00	3.23	3.23
9	onvolvulace	*C.arvensis*	3.00	3.00	100.00	100.00	3.23	3.23
10	uphorbiace	*E.peplus*	2.00	1.00	100.00	100.00	3.23	3.23
11	Fabaceae	M.indica	1.00	1.00	100.00	100.00	3.23	3.23
12	Fabaceae	M. siculus	2.16	1.58	100.00	100.00	3.23	3.23
13	Fabaceae	*sium sativu*	3.00	2.00	100.00	100.00	3.23	3.23
14	Fabaceae	V. hirsuta	3.00	2.00	100.00	100.00	3.23	3.23
15	Fabaceae	*Vicia sativa*	3.00	2.00	100.00	100.00	3.23	3.23
16	Malvaceae	Л. parviflor	2.00	0.00	100.00	100.00	3.23	3.23
17	Poaceae	A. fatua	3.00	0.00	100.00	100.00	3.23	3.23
18	Poaceae	*B. tectorum*	1.50	1.25	100.00	100.00	3.23	3.23
19	Poaceae	Phalaris sp	2.00	1.68	100.00	100.00	3.23	3.23
20	Poaceae	*P. annua*	1.00	0.00	100.00	100.00	3.23	3.23
21	Poaceae	H.marinum	1.27	1.00	100.00	100.00	3.23	3.23
22	Poaceae	*Eragrostis*	1.00	1.00	100.00	100.00	3.23	3.23
23	Poaceae	E.colonum	1.50	1.00	100.00	100.00	3.23	3.23
24	Poaceae	L. perenne	2.00	1.00	100.00	100.00	3.23	3.23
25	Poaceae	C.ciliaris	1.50	1.00	100.00	100.00	3.23	3.23
26	Poaceae	*C.dactylon*	1.50	0.75	100.00	100.00	3.23	3.23
27	'olygonacea	E.spinosua	2.00	1.00	100.00	100.00	3.23	3.23
28	'olygonacea	*R. dentatus*	2.50	1.25	100.00	100.00	3.23	3.23
29	Plantagin	antago maj	1.00	1.00	100.00	100.00	3.23	3.23
30	Plantagin	Veronica	0.00	8.00	100.00	100.00	3.23	3.23
31	Urticaceae	U.urens	3.00	3.00	100.00	100.00	3.23	3.23
			59.80	42.38	3100.00	3100.00		

10/10 ^oC	10/15 ^oC	10/20 ^oC	10/25 ^oC	10/30 ^oC	10/35 ^oC	10/40 ^oC	15/15 ^oC	15/20 ^oC	15/25 ^oC
0.000	0.000	0.000	0.000	0.000	0.000	0.000	0.000	0.000	0.000
0.000	0.000	0.000	0.000	0.000	0.000	0.000	0.000	0.000	0.000
0.000	0.000	5.000	0.000	0.000	0.000	0.000	3.000	25.500	26.500
0.000	24.000	69.000	10.500	15.000	0.000	0.000	67.000	110.000	84.500
6.500	84.500	152.500	59.000	78.500	0.000	0.000	146.500	202.000	147.000
31.500	156.500	242.500	135.000	157.000	35.500	0.000	228.000	294.500	212.500
99.500	237.500	333.500	218.500	239.000	113.500	0.000	311.000	388.000	279.000
177.000	320.000	425.000	304.500	321.500	196.000	0.000	398.000	481.500	346.500
255.500	403.000	516.500	391.500	405.500	279.500	0.000	485.000	575.000	414.000
339.500	486.500	608.000	479.500	489.500	364.000	0.000	572.000	668.500	487.500
424.500	570.000	699.500	568.000	574.000	449.500	0.000	659.000	762.000	561.000
510.500	654.000	791.000	657.000	659.000	535.500	0.000	746.000	855.500	634.500
596.500	738.000	882.500	746.000	744.000	621.500	0.000	833.500	949.000	708.000
682.500	822.000	974.000	835.000	829.000	707.500	0.000	921.000	1042.500	781.500
770.000	907.500	1067.000	925.500	915.500	795.000	1.500	1010.000	1137.500	856.500
858.000	993.500	1160.500	1016.500	1002.500	883.000	3.500	1099.500	1233.000	932.000
946.500	1079.500	1254.000	1107.500	1089.500	971.000	5.500	1189.000	1328.500	1007.500
1035.000	1165.500	1347.500	1198.500	1176.500	1059.000	7.500	1278.500	1424.000	1083.000
1123.500	1251.500	1441.000	1289.500	1263.500	1147.000	9.500	1368.000	1519.500	1158.500
1212.000	1337.500	1534.500	1380.500	1350.500	1235.000	11.500	1457.500	1615.000	1234.000
1300.500	1423.500	1628.000	1471.500	1437.500	1323.000	13.500	1547.000	1710.500	1309.500
1389.000	1509.500	1721.500	1562.500	1524.500	1411.000	15.500	1636.500	1806.000	1385.000
1477.500	1595.500	1815.000	1653.500	1611.500	1499.000	17.500	1726.000	1901.500	1460.500
1566.000	1681.500	1908.500	1744.500	1698.500	1587.000	19.500	1815.500	1997.000	1536.000
1654.500	1767.500	2002.000	1835.500	1785.500	1675.000	21.500	1905.000	2092.500	1611.500
1743.000	1853.500	2095.500	1926.500	1872.500	1763.000	23.500	1994.500	2188.000	1687.000
1831.500	1939.500	2189.000	2017.500	1959.500	1851.000	25.500	2084.000	2283.500	1762.500
1920.000	2025.500	2282.500	2108.500	2046.500	1939.000	27.500	2173.500	2379.000	1838.000
2008.500	2111.500	2376.000	2199.500	2133.500	2027.000	29.500	2263.000	2474.500	1913.500
2097.000	2197.500	2469.500	2290.500	2220.500	2115.000	31.500	2352.500	2570.000	1989.000

Fresh Leaves	dry Leaves	Fresh Fruit	dry Fruit	whole	whole	Leav aarea
0.03	0.0016	0.00	0.00	0.52	0.092	1.50
0.03	0.0016	0.00	0.00	0.47	0.0833	4.70
0.03	0.0016	0.00	0.00	0.43	0.0832	4.16
0.03	0.0016	0.00	0.00	0.47	0.086167	4.29
0.03	0.0016	0.00	0.00	0.47	0.086167	3.66
0.29	0.040	0.00	0.00	3.86	1.129	7.00
0.28	0.031	0.00	0.00	3.99	1.087	7.70
0.30	0.033	0.00	0.00	3.78	1.0976	7.16
0.29	0.035	0.00	0.00	3.88	1.104533	7.29
0.29	0.035	0.00	0.00	3.88	1.104533	7.29
0.39	0.165	0.00	0.00	5.59	1.853	8.00
0.38	0.161	0.00	0.00	4.96	1.956	8.50

0.40	0.176	0.00	0.00	5.10	1.843	8.76
0.39	0.167	0.00	0.00	5.22	1.884	8.42
0.39	0.167	0.00	0.00	5.22	1.884	8.42
0.46	0.1857	0.05	0.00252	7.20	2.687	8.13
0.35	0.185	0.04	0.00262	7.98	2.675	8.16
0.54	0.196	0.039	0.00258	8.20	2.765	8.35
0.45	0.189	0.043	0.002573	7.79	2.709	8.22
0.45	0.189	0.043	0.002573	7.79	2.709	8.22
0.46	0.1857	0.05	0.00252	7.20	2.687	8.13
0.35	0.185	0.04	0.00262	7.98	2.675	8.16
0.54	0.196	0.039	0.00258	8.20	2.765	8.35
0.45	0.189	0.043	0.002573	7.79	2.709	8.22
0.45	0.19	0.043	0.002573	7.79	2.71	8.22

					Dry mass of leaves, st		Leaf dry m	Leaf dry m	Root dry m
Length	number of	Area	Leaf dry m	Stem dry n	Total dry n	Leaf area ratio		Leaf mass	Root mass
3.333	2.660	2.233	0.002	0.070	0.135	44.02		0.019	0.735
7.667	11.667	8.286	0.035	0.470	0.720	134.34		0.031	0.195
15.250	28.333	8.420	0.167	0.960	1.459	163.51		0.089	0.176
18.833	45.000	8.608	0.189	1.391	2.157	179.59		0.070	0.213
18.830	40.000	8.600	0.199	1.533	2.422	142.04		0.063	0.233
		0.74031	0.991806	0.954348	0.944274	0.755		0.787	0.684

Relative Growth Rate (mg. days)

Leaves	Fruit	Total dry biomass
0.05	0.00	49.83
0.31	0.00	17.88
-0.26	0.00	7.97
-0.01	0.00	3.22
19.61	0.03	1.93

mg mm^{-2} day^{-1}

Leaves	Fruit	Total dry biomass
0.00	0.00	0.28
0.00	0.00	0.99
0.00	0.00	-0.29
0.00	6.78	-0.01
0.60	73.74	28.25

field uni	Relative density		Frequency		Relative frequency (R	Abundance		Relative Al	
Non Inf.	Infected	Non Inf.	Infected	Non Inf.	Infected	Non Inf.	Infected	Non Inf.	Infected
6.11	2.98	1.00	1.00	0.04	0.04	0.03	1.00	10.21	10.09
6.11	2.98	1.00	1.00	0.04	0.04	3.00	1.00	16.65	10.09
1.67	0.00	1.00	1.00	0.04	0.04	0.82	0.00	7.49	4.04
4.07	2.98	1.00	1.00	0.04	0.04	2.00	1.00	12.45	10.09
4.07	2.98	1.00	1.00	0.04	0.04	2.00	1.00	12.45	10.09
4.40	2.98	1.00	1.00	0.04	0.04	2.16	1.00	13.11	10.09
4.07	5.96	1.00	1.00	0.04	0.04	2.00	2.00	12.45	16.15
3.87	4.25	1.00	1.00	0.04	0.04	1.90	1.43	12.03	12.67
2.04	0.00	1.00	1.00	0.04	0.04	1.00	0.00	8.24	4.04
4.88	3.47	1.00	1.00	0.04	0.04	2.40	1.17	14.12	11.09
6.47	5.96	1.00	1.00	0.04	0.04	3.18	2.00	17.39	16.15
4.31	6.47	1.00	1.00	0.04	0.04	2.12	2.17	12.95	17.17
4.05	5.96	1.00	1.00	0.04	0.04	1.99	2.00	12.41	16.15
4.07	2.98	1.00	1.00	0.04	0.04	2.00	0.00	12.45	7.02
4.07	2.98	1.00	1.00	0.04	0.04	2.00	1.00	12.45	10.09
4.07	2.98	1.00	1.00	0.04	0.04	2.00	1.00	12.45	10.09
6.11	5.96	1.00	1.00	0.04	0.04	3.00	2.00	16.65	16.15
1.59	0.00	1.00	1.00	0.04	0.04	0.78	0.00	7.33	4.04
2.50	2.85	1.00	1.00	0.04	0.04	1.23	0.96	9.20	9.83
3.47	3.44	1.00	1.00	0.04	0.04	1.70	1.15	11.20	11.02
3.97	5.01	1.00	1.00	0.04	0.04	1.95	1.68	12.23	14.22
6.11	8.94	1.00	1.00	0.04	0.04	3.00	3.00	16.65	22.20
3.84	0.00	1.00	1.00	0.04	0.04	1.88	0.00	11.96	4.04
0.00	11.92	1.00	1.00	0.04	0.04	0.00	4.00	4.04	28.25
4.07	5.96	1.00	1.00	0.04	0.04	2.00	2.00	12.45	16.15
100.00	100.00	25.00	25.00	1	1	46.13861	32.55077	301	301

field uni	Relative density		Frequency		Relative frequency (R	Abundance		Relative Al	
Non Inf.	Infected	Non Inf.	Infected	Non Inf.	Infected	Non Inf.	Infected	Non Inf.	Infected
3.34	2.36	1.00	1.00	0.03	0.03	0.02	0.01	10.06	8.24
5.02	2.36	1.00	1.00	0.03	0.03	0.03	0.00	13.46	5.62

3.18	2.36	1.00	1.00	0.03	0.03	0.00	0.01	6.44	8.24
3.09	0.00	1.00	1.00	0.03	0.03	0.02	0.00	9.54	3.26
3.34	2.36	1.00	1.00	0.03	0.03	0.02	0.01	10.06	8.24
3.24	2.36	1.00	1.00	0.03	0.03	0.02	0.01	9.84	8.24
3.34	2.36	1.00	1.00	0.03	0.03	0.02	0.01	10.06	8.24
1.97	2.04	1.00	1.00	0.03	0.03	0.01	0.01	7.27	7.57
5.02	7.08	1.00	1.00	0.03	0.03	0.03	0.03	13.46	18.21
3.34	2.36	1.00	1.00	0.03	0.03	0.02	0.01	10.06	8.24
1.67	2.36	1.00	1.00	0.03	0.03	0.01	0.00	6.66	5.62
3.61	3.73	1.00	1.00	0.03	0.03	0.02	0.02	10.60	11.13
5.02	4.72	1.00	1.00	0.03	0.03	0.03	0.02	13.46	13.22
5.02	4.72	1.00	1.00	0.03	0.03	0.03	0.02	13.46	13.22
5.02	4.72	1.00	1.00	0.03	0.03	0.03	0.02	13.46	13.22
3.34	0.00	1.00	1.00	0.03	0.03	0.02	0.00	10.06	3.26
5.02	0.00	1.00	1.00	0.03	0.03	0.03	0.00	13.46	3.26
2.51	2.95	1.00	1.00	0.03	0.03	0.02	0.00	8.36	6.21
3.34	3.97	1.00	1.00	0.03	0.03	0.02	0.02	10.06	11.65
1.67	0.00	1.00	1.00	0.03	0.03	0.01	0.00	6.66	3.26
2.12	2.36	1.00	1.00	0.03	0.03	0.01	0.01	7.58	8.24
1.67	2.36	1.00	1.00	0.03	0.03	0.01	0.01	6.66	8.24
2.51	2.36	1.00	1.00	0.03	0.03	0.02	0.00	8.36	5.62
3.34	2.36	1.00	1.00	0.03	0.03	0.02	0.01	10.06	8.24
2.51	2.36	1.00	1.00	0.03	0.03	0.02	0.01	8.36	8.24
2.51	1.77	1.00	1.00	0.03	0.03	0.02	0.01	8.36	6.99
3.34	2.36	1.00	1.00	0.03	0.03	0.02	0.01	10.06	8.24
4.18	2.95	1.00	1.00	0.03	0.03	0.03	0.01	11.76	9.49
1.67	2.36	1.00	1.00	0.03	0.03	0.01	0.01	6.66	8.24
0.00	18.88	1.00	1.00	0.03	0.03	0.00	0.08	3.26	43.12
5.02	7.08	1.00	1.00	0.03	0.03	0.03	0.03	13.46	18.21
100.00	100.00	31.00	31.00	1.00	1.00	0.58	0.38	301.00	301.00

15/30 °C	15/35 °C	15/40 °C	20/20 °C	20/25 °C	20/30 °C	20/35 °C	20/40 °C	25/25 °C	25/30 °C
0.000	0.000	0.000	0.000	0.000	0.000	0.000	0.000	0.000	0.000
0.000	0.000	0.000	7.500	3.500	3.000	0.000	0.000	13.087	7.500
41.500	0.000	0.000	71.500	72.500	71.500	6.000	0.000	80.726	64.500
121.000	50.000	0.000	142.500	158.500	154.500	74.000	11.500	156.416	131.000
206.500	124.000	0.000	215.500	249.000	239.500	144.000	39.000	237.190	198.500
295.000	201.500	0.000	288.500	340.500	325.000	214.500	81.500	319.006	267.000
384.500	282.000	0.500	361.500	432.500	411.000	285.500	128.500	403.404	335.500
474.000	365.000	6.000	435.500	525.000	497.500	357.500	190.500	487.803	404.000
563.500	448.000	18.500	510.000	617.500	584.000	430.500	255.000	572.202	472.500
653.000	531.000	33.000	584.500	710.000	670.500	504.000	321.500	656.600	541.000
743.000	615.000	61.500	659.000	802.500	757.000	577.500	389.500	740.999	609.500
833.000	699.000	91.000	733.500	895.500	843.500	651.000	457.500	825.398	678.000
923.000	783.000	126.000	808.000	988.500	930.000	724.500	525.500	909.796	746.500
1013.000	867.000	162.500	882.500	1081.500	1016.500	798.000	593.500	994.195	815.000
1104.500	952.500	200.500	958.500	1176.000	1104.500	873.000	663.000	1080.135	885.000
1196.500	1038.500	239.000	1035.000	1271.000	1193.000	948.500	733.000	1166.565	955.500
1288.500	1124.500	277.500	1111.500	1366.000	1281.500	1024.000	803.000	1252.995	1026.000
1380.500	1210.500	316.500	1188.000	1461.000	1370.000	1099.500	873.000	1339.426	1096.500
1472.500	1296.500	355.500	1264.500	1556.000	1458.500	1175.000	943.000	1425.856	1167.000
1564.500	1382.500	394.500	1341.000	1651.000	1547.000	1250.500	1013.000	1512.286	1237.500
1656.500	1468.500	433.500	1417.500	1746.000	1635.500	1326.000	1083.000	1598.716	1308.000
1748.500	1554.500	472.500	1494.000	1841.000	1724.000	1401.500	1153.000	1685.146	1378.500
1840.500	1640.500	511.500	1570.500	1936.000	1812.500	1477.000	1223.000	1771.576	1449.000
1932.500	1726.500	550.500	1647.000	2031.000	1901.000	1552.500	1293.000	1858.006	1519.500
2024.500	1812.500	589.500	1723.500	2126.000	1989.500	1628.000	1363.000	1944.437	1590.000
2116.500	1898.500	628.500	1800.000	2221.000	2078.000	1703.500	1433.000	2030.867	1660.500
2208.500	1984.500	667.500	1876.500	2316.000	2166.500	1779.000	1503.000	2117.297	1731.000
2300.500	2070.500	706.500	1953.000	2411.000	2255.000	1854.500	1573.000	2203.727	1801.500
2392.500	2156.500	745.500	2029.500	2506.000	2343.500	1930.000	1643.000	2290.157	1872.000
2484.500	2242.500	784.500	2106.000	2601.000	2432.000	2005.500	1713.000	2376.587	1942.500

Stem dry n	Leaf area⁄	Stem lengt)1		
Stem mass	Specific lea	Specific ste	S⁄ R ratio	Shoot to root ratio
0.812	1370.70	47.62	1.131	
0.425	238.79	16.33	2.342	
0.509	50.32	15.89	3.395	
0.514	45.58	13.54	2.740	
0.510	43.25	12.28	2.466	
0.477	0.968	0.74	0.667	

bundance		Relative Abundance
	Uninvaded	Invaded
S.oleraceus	10.12	10.09
C. bursa-pastc	16.78	10.09
C.endivia.	7.53	4.04
S. marianun	12.53	10.09
Sisymbriur	12.53	10.09
Chenopodium album	13.21	10.09
C.arvensis	12.53	16.15
E.peplus	12.11	12.67
M.indica	8.29	4.04
M. siculus	14.22	11.09
Pisium sativiu	17.53	16.15
T. tomentosu	13.04	17.17
Vicia sativa	12.49	16.15
M. parviflor	12.53	7.02
B. tectorum	12.53	10.09
Phalaris sp	12.53	10.09
P. annua	16.78	16.15
H.marinum	7.36	4.04
E.colonum	9.25	9.83
L. perenne	11.28	11.02
C.ciliaris	12.31	14.22
C.dactylon	16.78	22.20
Plantago maj	12.04	4.04
Veronica	4.04	28.25
U.urens	12.53	16.15

bundance		Relative Abundance
	Uninvaded	Invaded
S.oleraceus	10.06	8.24
C. bursa-pastc	13.46	5.62

C.endivia.	6.44	8.24
C.bonariensi	9.54	3.26
0 S. marianun	10.06	8.24
Sisymbriur	9.84	8.24
B.vulgris	10.06	8.24
Chenopo dium album	7.27	7.57
C.arvensi s	13.46	18.21
E.peplus	10.06	8.24
M.indica	6.66	5.62
M. siculus	10.60	11.13
Pisium sativiu	13.46	13.22
T. tomentosu	13.46	13.22
Vicia sativa	13.46	13.22
M. parviflor	10.06	3.26
A. fatua	13.46	3.26
B. tectorum	8.36	6.21
Phalaris sp	10.06	11.65
P. annua	6.66	3.26
H.marinum	7.58	8.24
Eragrostis	6.66	8.24
E.colonum	8.36	5.62
L. perenne	10.06	8.24
C.ciliaris	8.36	8.24
C.dactylo n	8.36	6.99
E.spinosua	10.06	8.24
R. dentatus	11.76	9.49
Plantago maj	6.66	8.24
Veronica	3.26	43.12
U.urens	13.46	18.21

25/35 °C	25/40 °C	30/30 °C	30/35 °C	30/40 °C	35/35 °C	35/40 °C	40/40 °C
0.000	0.000	0.000	0.000	0.000	0.000	0.000	0.000
2.000	0.000	0.000	0.000	0.000	0.000	0.000	0.000
58.000	0.000	20.000	53.500	0.000	0.000	0.000	0.000
124.500	0.000	41.000	111.000	0.000	0.000	0.000	0.000
195.000	0.000	62.500	169.000	5.500	1.500	0.000	0.000
266.500	2.500	84.500	228.000	18.500	4.000	0.000	0.000
338.000	12.000	109.000	287.000	32.500	8.000	0.000	0.000
409.500	22.000	134.500	347.000	46.500	14.000	0.000	0.000
481.000	33.000	160.000	407.000	61.000	20.500	0.000	0.000
552.500	45.500	185.500	467.000	76.000	28.500	1.000	0.000
624.000	58.000	211.000	527.000	91.000	37.000	2.500	0.000
695.500	71.000	236.500	587.000	106.000	46.000	4.500	0.000
767.000	84.000	262.000	647.000	121.000	56.000	7.500	0.000
838.500	97.000	287.500	707.000	136.000	66.000	11.000	0.000
911.500	111.500	314.500	768.500	152.500	77.000	16.000	0.000
985.000	126.500	342.000	830.500	169.500	88.500	21.500	0.000
1058.500	141.500	369.500	892.500	186.500	100.500	27.000	0.000
1132.000	156.500	397.000	954.500	203.500	113.000	32.500	0.000
1205.500	171.500	424.500	1016.500	220.500	125.500	38.500	0.000
1279.000	186.500	452.000	1078.500	237.500	138.500	45.500	0.000
1352.500	201.500	479.500	1140.500	254.500	151.500	52.500	0.000
1426.000	216.500	507.000	1202.500	271.500	164.500	59.500	0.000
1499.500	231.500	534.500	1264.500	288.500	177.500	66.500	0.000
1573.000	246.500	562.000	1326.500	305.500	191.000	73.500	0.000
1646.500	261.500	589.500	1388.500	322.500	204.500	80.500	0.000
1720.000	276.500	617.000	1450.500	339.500	218.000	87.500	0.000
1793.500	291.500	644.500	1512.500	356.500	231.500	94.500	0.000
1867.000	306.500	672.000	1574.500	373.500	245.000	101.500	0.000
1940.500	321.500	699.500	1636.500	390.500	258.500	108.500	0.000
2014.000	336.500	727.000	1698.500	407.500	272.000	115.500	0.000
